# Development of a S-adenosylmethionine analog that intrudes the RNA-cap binding site of Zika methyltransferase

**DOI:** 10.1038/s41598-017-01756-7

**Published:** 2017-05-09

**Authors:** Rinku Jain, Kyle V. Butler, Javier Coloma, Jian Jin, Aneel K. Aggarwal

**Affiliations:** Department of Pharmacological Sciences, Icahn School of Medicine at Mount Sinai, 1425 Madison Avenue, New York, New York USA

## Abstract

The Zika virus (ZIKV) has emerged as a major health hazard. We present here a high resolution structure (1.55 Å) of ZIKV NS5 methyltransferase bound to a novel S-adenosylmethionine (SAM) analog in which a 4-fluorophenyl moiety substitutes for the methyl group. We show that the 4-fluorophenyl moiety extends into a portion of the RNA binding tunnel that typically contains the adenosine 2′OH of the RNA-cap moiety. Together, the new SAM analog and the high-resolution crystal structure are a step towards the development of antivirals against ZIKV and other flaviviruses.

## Introduction

The Zika virus (ZIKV) belongs to the *Flavivirus* genus that includes mosquito-borne human pathogens such as dengue virus (DENV1-4), Murray Valley encephalitis virus (MVEV), West Nile virus (WNV), yellow fever virus (YFV), and Japanese encephalitis virus (JEV), among others^1^. ZIKV has emerged as a major health concern over the past year due to its proclivity to infect nerves *in utero* and in adults, leading to microcephaly in newborn infants and the Guillan-Barré syndrome in adults, respectively^2, 3^. Additional modes of transmission such as sexual transmission^4^ and blood transfusions^5^ threaten to hasten the spread of the virus. In addition to efforts to eradicate the *Aedes* mosquito vectors, there is an urgent need to develop effective vaccines and antiviral agents against ZIKV. One approach to the discovery of antivirals is to target enzymes that are essential for the replication and survival of the virus.

A methylated 5′-cap is a key feature of both flavivirus genomic RNA and host messenger RNA, required for RNA stability and translation^6^. In flaviviruses, the 5′-cap (^N7Me^GpppA_2′OMe_; where Me is a methyl group) is formed by the activities of two enzymes: NS3 that encodes a protease and helicase, and NS5 that encodes a methyltransferase (NS5-MTase) and an RNA dependent RNA polymerase. The NS5-MTase domain methylates both the N7 and 2′O atoms in a sequential reaction: GpppA_2′OH_-RNA → ^N7Me^GpppA_2′OH_-RNA or “cap-0” → ^N7Me^GpppA_2′OMe_-RNA or “cap-1”, using S-adenosylmethionine (SAM) as the methyl donor and generating S-adenosylhomocysteine (SAH) as the reaction byproduct^6^.

Mutations in NS5-MTase that lead to defects in N7 methylation are lethal in flaviviruses^7–9^. Defects in 2′O methylation attenuate the virus and is a basis for vaccine development^10, 11^. Together, these features make ZIKV NS5-MTase an attractive target for the development of antivirals. We present here the design and characterization of a novel SAM analog (MS2042) with a 4-fluorophenyl moiety. We determine the thermodynamics parameters of binding of MS2042 to ZIKV NS5-MTase and elucidate a high-resolution (1.55 Å) structure of MS2042 bound to ZIKV NS5-MTase (NS5-MTase_MS2042_). We show that the 4-fluorophenyl moiety extends into a portion of the RNA binding tunnel that typically contains the adenosine 2′OH of the cap-0 structure (^N7Me^Gppp**A**_**2′OH**_-RNA). Together, the new SAM analog and the high-resolution crystal structure provide a path for further drug development.

## Results

### Design and synthesis of MS2042

We designed MS2042 on the basis of recent high resolution structures of the ZIKV NS5-MTase bound to SAM alone (NS5-MTase_SAM_), or to both SAM and the 5′cap mimic 7-methyl guanosine diphosphate (7-MeGpp, NS5-MTase_SAM,7-MeGpp_)^12^. We postulated that a 4-fluorophenyl group attached to the Cε atom of the methionine portion of SAM would have the capacity to bind a portion of the RNA binding tunnel that spans the SAM and 7-MeGpp binding sites. Supplementary Fig. 1a shows the scheme for the synthesis of MS2042.

### Thermodynamic parameters of MS2042 binding to ZIKV NS5-MTase

**Figure 1:**
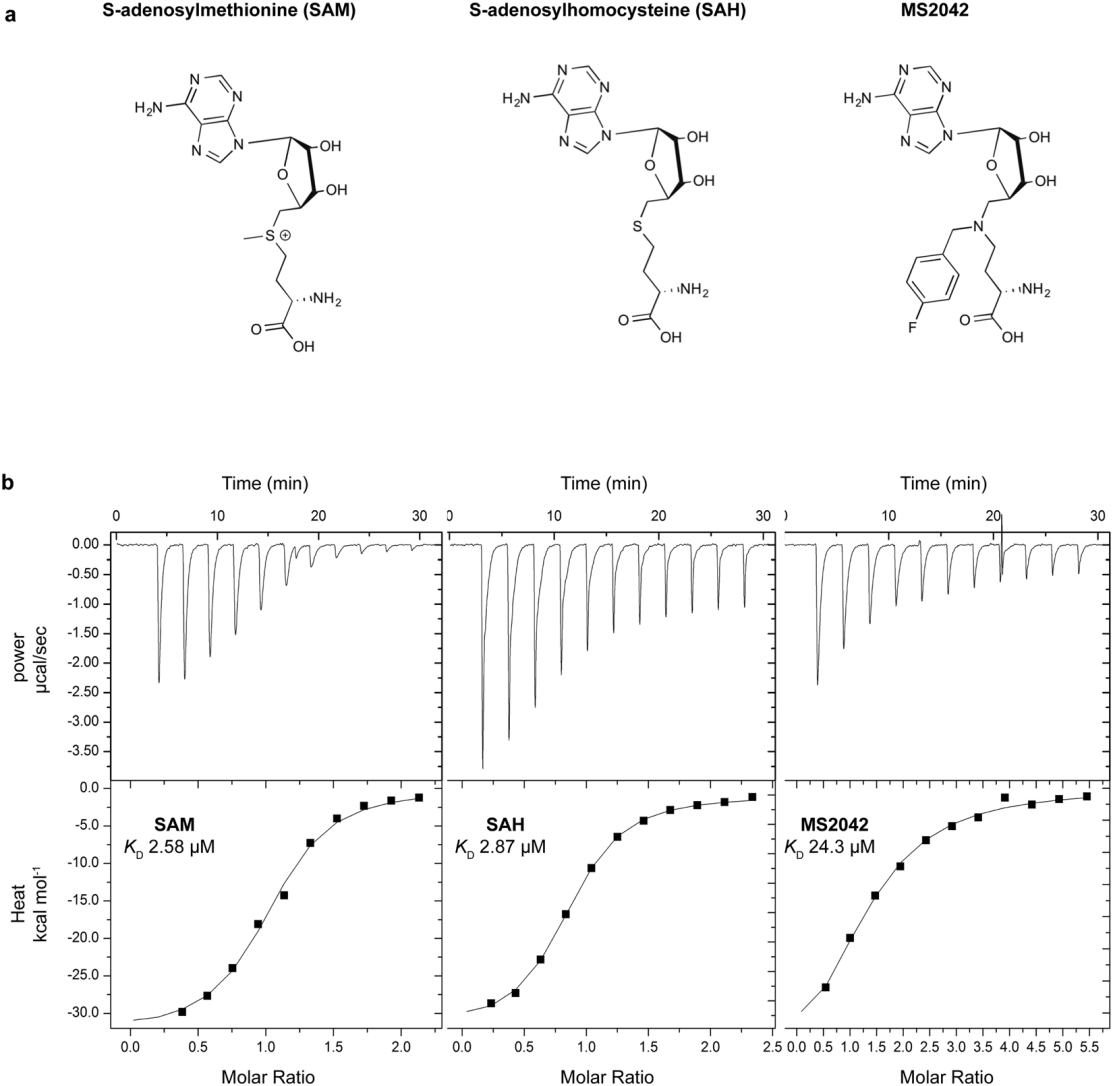
(**a**) Chemical structures and (**b**) Raw isothermal titration calorimetry data and binding isotherms for SAM (left), SAH (middle), and MS2042 (right) binding to ZIKV NS5-MTase.

A challenge in determining the binding of MS2042 to ZIKV NS5-MTase is in the preparation of the apo protein. This is because ZIKV NS5-MTase (residues 1–266) purifies from *E*. *coli* with SAM/SAH bound at the active site (NS5-MTase_SAM_). To remove the bound SAM/SAH, we first denatured NS5-MTase_SAM_ with 8 M urea and then refolded and purified the protein by dialysis and size exclusion chromatography (Supplementary Fig. 1b). We used isothermal titration calorimetry (ITC) to determine and compare the thermodynamic parameters of SAM, SAH, and MS2042 binding to refolded ZIKV NS5-MTase (Fig. 1). The enzyme binds SAM and SAH with similar affinities and equilibrium dissociation constant *K*_D_ values of 2.58 μM and 2.87 μM (Table 1), respectively. By comparison, the enzyme binds MS2042 with a *K*_D_ of 24.3 μM. Thus, the ZIKV enzyme can effectively bind MS2042 with its extra 4-fluorophenyl group, with an affinity that is ~9.4 lower than SAM (Table 1). To elucidate the structural determinants of MS2042 binding and how it compares to that of SAM, we determined the structure of MS2042 bound to ZIKV NS5-MTase (NS5-MTase_MS2042_).

**Table 1:** .

Ligand	*K*_D_ (μM)	ΔG (Kcal mol^−1^)	ΔH (Kcal mol^−1^)	−TΔS (Kcal mol^−1^)	*K*_D_ relative to that of SAM
SAM	2.58	−7.70	−32.6	24.9	—
SAH	2.87	−7.60	−39.8	32.2	1.11
MS2042	24.3	−6.30	−20.3	14.0	9.42

Thermodynamic binding parameters of SAM, SAH, and MS2042 to ZIKV NS5-MTase.

### Overall structure of ZIKV NS5-MTase bound to MS2042

**Figure 2:**
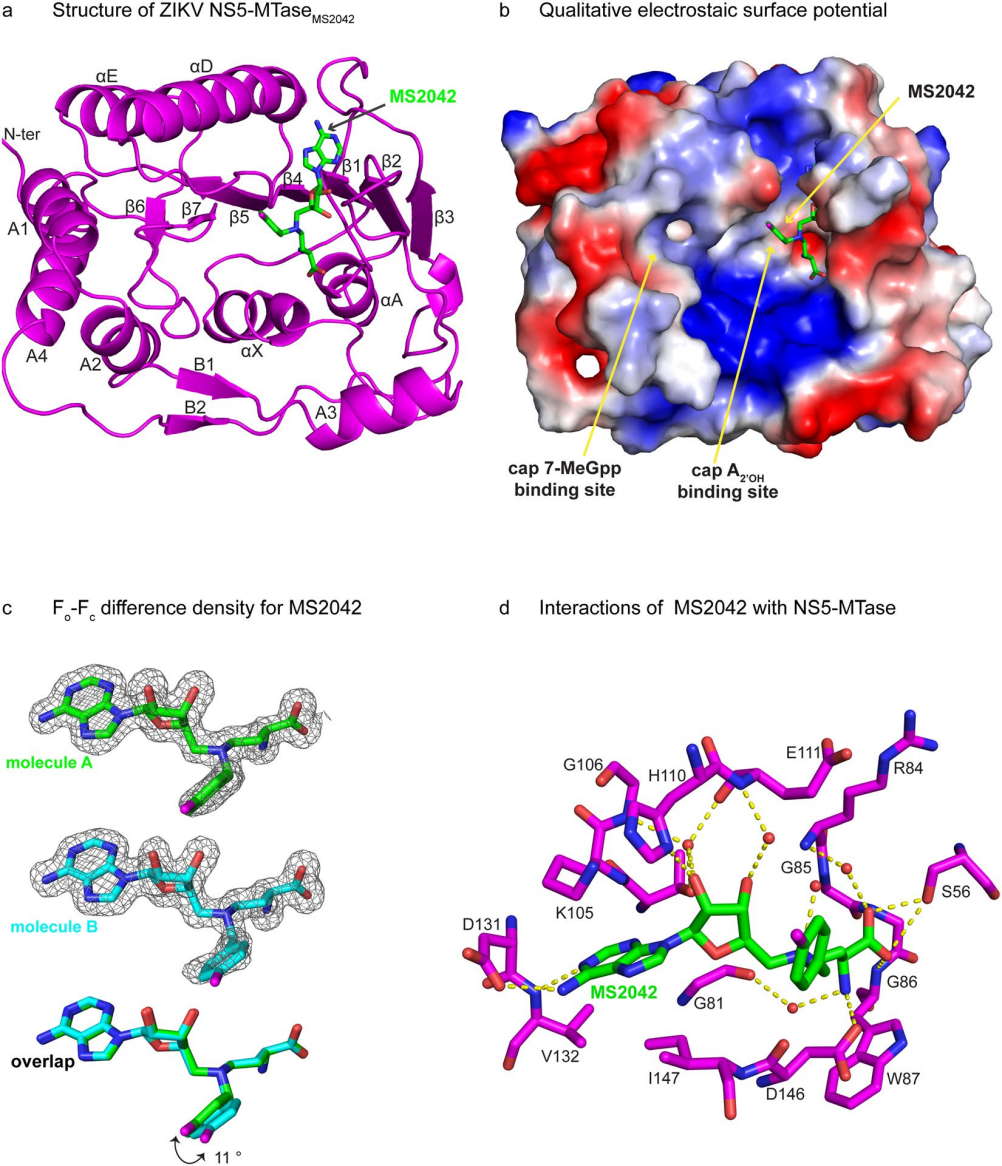
Structure of NS5-MTase_MS2042_. (**a**) Overall structure of NS5-MTase_MS2042_ with labeled secondary structures. (**b**) Qualitative electrostatic surface potential of NS5-MTase_MS2042_ showing the shallow cavity accommodating the 4-fluorophenyl group of MS2042. The SAM portion of MS2042 is partially hidden from view (**c**) F_o_-F_c_ difference density over MS2042 contoured at 2.5σ. Superimposition of MS2042 bound to molecules A and B reveals a slight difference in the orientation of the 4-fluorophenyl groups. (**d**) Interactions of MS2042 with the protein chain. Hydrogen bonds and solvent molecules are depicted as dashed lines and red spheres, respectively.

For crystallization, NS5-MTase and MS2042 were mixed in a 1:10 molar ratio. NS5-MTase_MS2042_ crystallized from solution containing PEG 4000, 2-propanol, sodium citrate tribasic and glycerol. Crystals belong to space group P2(1) with unit cell dimensions of a = 38.99 Å, b = 111.23 Å, c = 77.40 Å, and β = 93.9° (Supplementary Table 1). The structure of NS5-MTase_MS2042_ was determined by molecular replacement using the protein chain of NS5-MTase_SAM,7-MeGpp_ (PDB id: 5KQS) as the starting model, and refined to 1.55 Å resolution with R_work_ and R_free_ values of 17.4% and 19.9%, respectively (Supplementary Table 1). The asymmetric unit contains two molecules of NS5-MTase (molecules A and B) related by two fold non-crystallographic symmetry (Supplementary Fig. 2). The two molecules are nearly identical and superimpose with an r.m.s deviation of 0.12 Å for 254 main chain Cα atoms. Changes are limited mainly to the side chain conformations of solvent exposed loop regions, including amino acids 108–110 (adjacent to the SAM binding cavity) and amino acids 253–255 that mediate contacts between the two molecules in the asymmetric unit. Overall, the core of ZIKV NS5-MTase_MS2042_ adopts a Rossmann fold (residues 54–223) with seven β-strands (β1-β7) and four α-helices (αX, αA, αD, αE). The core is flanked by α-helices (A1-A4) and β-strands (B1-B2) that form the N- and C-terminal extensions and cradle the core (Fig. 2a). Amino acids belonging to the catalytic tetrad (Lys_61_-Asp_146_-Lys_182_-Glu_218_; a feature of 2′O methyltransferases) adopt the same conformation as in ZIKV NS5-MTase_SAM_ and NS5-MTase_SAM,7-MeGpp_ structures. Overall, the NS5-MTase_MS2042_ structure is very similar to the NS5-MTase_SAM_ and NS5-MTase_SAM,7-MeGpp_ structures with r.m.s deviations of 0.17 Å (247 Cα atoms) and 0.18 Å (254 Cα atoms) respectively, indicative of little or no change in the protein conformation in accommodating the extra 4-fluorophenyl group of MS2042 (Supplementary Fig. 2b)^12^.

### Mode of MS2042 binding

The electron density for MS2042 is clear in both molecules of the asymmetric unit (Fig. 2c). The SAM portion of MS2042 overlays almost exactly with the SAM in the NS5-MTase_SAM_ and NS5-MTase_SAM,7-MeGpp_ structures (Supplementary Fig. 2c), wherein the adenine ring is sandwiched between the side chains of Lys105 (partially disordered for chain B) and Ile147, and the N1 and N6 atoms establish hydrogen bonds with the backbone amide of Val132 and the side chain of Asp131 (Fig. 2d). In addition, the ribose sugar participates in solvent mediated interactions with Gly106, Glu111 and Thr104, while the amino and carboxyl groups of the methionine portion are held in place by hydrogen bonds with the side chains of Ser56 and Asp146, and the backbone amides of Gly86 and Trp87. A minor difference between molecules A and B in the NS5-MTase_MS2042_ structure is that whereas the 2′OH of the MS2042 ribose in molecule A interacts with the side chain of His110, in molecule B His110 is pushed away into the solvent, in a conformation similar to that in the SAM bound structures (Supplementary Fig. 2c).

**Figure 3:**
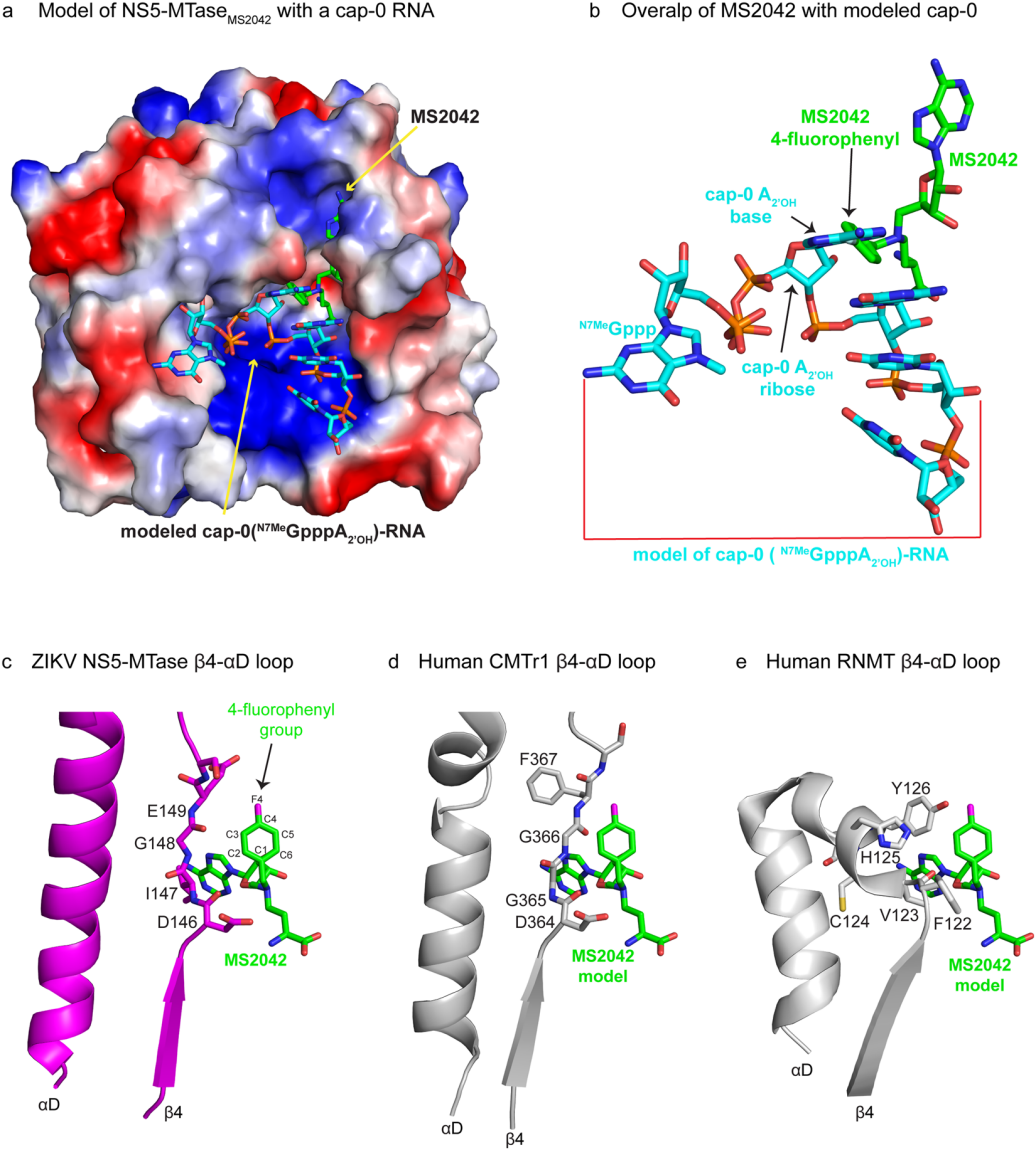
Model of ZIKV NS5-MTase_MS2042_ with cap-0 RNA and comparison with  the human mRNA cap methyltransferases CMTr1 and RNMT. (**a**) Qualitative electrostatic surface potential of ZIKV NS5-MTase_MS2042_ with a cap-0(^N7Me^GpppA_2′OH_) RNA model derived from the structure of DENV3 NS5 (5DTO). (**b**) Magnified view of the model showing steric overlap of the cap-0 adenine and its 2′O atom with the 4-fluorophenyl group of MS2042. Comparison of loop β4-αD in (**c**) ZIKV NS5-MTase, (**d**) human nucleoside-2′-O methyltransferase (CMTr1, PDB id 4N49), and (**e**) human mRNA cap guanine-N7 methyltransferase (RNMT, PDB id 3EPP).

The 4-fluorophenyl group of MS2042 extends into a shallow cavity, adjacent to the SAM binding pocket (Fig 2b). The orientation of the phenyl ring differs slightly between the two molecules due to an ~11° rotation about the Nδ-Cε bond of SAM (Fig. 2c). There are no specific hydrogen bonds with any amino acids and the fluorine atom projects into the solvent. Most of the contacts between the 4-fluorophenyl group and the NS5-MTase are van der Waals in nature, wherein one edge of the phenyl ring (C2-C3) runs parallel to amino acids (146-Asp-Ile-Gly-Glu-149) in the NS5-MTase loop connecting β4 and αD (Fig. 3c) of the Rossmann core. Most importantly, the shallow cavity in the which 4-fluorophenyl group lies is the putative binding site of the base and the 2′OH group of the cap-0 adenosine (^N7Me^Gpp**A**_**2′OH**_-RNA) when RNA binds a flavivirus NS5 methyltransferase. Figure 3 shows the cap-0 moiety (^N7Me^GppA_2′OH_) modeled in our NS5-MTase_MS2042_ structure, based on the structure of DENV3 NS5 harboring a cap-0 containing RNA substrate^13^. From our modeling, adenine base and the 2′O atom of the cap-0 structure sterically overlap with the phenyl ring of MS2042 and would be hindered in being methylated (Fig. 3a,b).

## Discussion

There has been a significant effort to identify small molecule inhibitors for flavivirus MTases^14–20^. Interestingly, a DENV-selective SAM/SAH analog was identified, in which a chlorophenyl moiety is attached the adenine N6 atom of SAM/SAH^9, 16^. The chlorophenyl moiety extends into a small hydrophobic cavity located near the adenine of SAM/SAH. Since this cavity is mutually exclusive to the cavity that binds the 2′OH of the cap-0 structure, SAM/SAH analogs with dual substituents (one on the N6 atom of adenine and the other on Cε atom of the methionine portion of SAM/SAH) may provide additional selectivity for flavivirus MTases. In this context, the 4-fluorophenyl group on MS2042 also provides a basis for further chemical modifications. In particular, one edge of the 4-fluorophenyl ring (C2-C3) (Fig. 3c) runs parallel to a stretch of amino acids (146-Asp-Ile-Gly-Glu-149) in the NS5-MTase loop connecting β4 and αD. Based on our modeling, an exocyclic OH at the C2 position and/or NH at the C3 position would have the capacity to interact directly with amino acids on this β4-αD loop. Moreover, this loop is conserved in all flavivirus MTases but is different in sequence and structure in the human nucleoside-2′-O methyltransferase (CMTr1, PDB id 4N49)^21^ and mRNA cap guanine-N7 methyltransferase (RNMT; PDB id 3EPP). The β4 -αD loop of human CMTr1 (364-Asp-Gly-Gly-Phe-367) and RNMT (122-Phe-Val-Cys-His-Tyr-126) is pushed up against the 4-fluorophenyl moiety of a modeled MS2042, relative to its location in ZIKV NS5-MTase (Fig. 3c–e and Supplementary Fig. 3). Based on our modeling, substituents on the C2, C3 and F4 positions of the 4-fluorophenyl moiety would be prone to steric clashes with the β4-αD loop of CMTr1 (Fig. 3d) and RNMT (Fig. 3e). Accordingly, MS2042 derivatives with chemotypes on the 4-fluorophenyl moiety offer the prospect of enhancing ligand selectivity for flavivirus NS5-MTases, relative to the human mRNA cap MTases.

In conclusion, we present here a high resolution structure of ZIKV NS5-MTase bound to a novel SAM analog as a step towards the development of antivirals against ZIKV and other pathogenic flaviviruses.

## Methods

### Purification of Apo ZIKV NS5-MTase

ZIKV NS5-MTase (1–266) from the H/PF/2013 strain was expressed and purified from *E*. *coli* strain LOBSTR (DE3) with a N-terminal His_6_-SUMO tag. Cell pellets containing the recombinant protein were resuspended in buffer containing 50% B-PER (Thermo Scientific), 25 mM Tris, pH 8.0, 500 mM NaCl, 5% glycerol, and 5 mM 2-mercaptoethanol (BME). Cells were lysed by sonication and the filtered lysate was loaded on a 5 mL Ni-NTA column (Qiagen). Protein bound to the Ni-NTA column was eluted with buffer containing 50 mM Tris-HCl, pH 8.0, 500 mM NaCl, 5% glycerol, 5 mM BME and 250 mM imidazole. Eluted protein was dialyzed into buffer containing 50 mM HEPES pH 7.5, 500 mM NaCl, 5% glycerol, and 5 mM BME. The His_6_-SUMO tag was cleaved with Ulp protease and the protein re-loaded on the Ni-NTA column to remove the cleaved His_6_-SUMO tag and any uncleaved protein. To remove SAM/SAH bound to the purified protein, the protein was denatured by adding urea to a final concentration of 8 M and overnight dialysis against buffer containing 50 mM Tris, 500 mM NaCl and 8 M urea. Refolding was initiated by dialyzing overnight into buffer containing 25 mM HEPES, pH 7.0, 250 mM NaCl, 2 mM TCEP, 5% glycerol, and 1 M urea. The refolded protein was purified further by size exclusion chromatography on a Superdex 75 16/600 GL column (GE Healthcare Life Sciences). Before crystallization, the protein was concentrated to 3.5 mg/ml in buffer containing 25 mM HEPES, pH 7.0, 250 mM NaCl, 5% glycerol and 2 mM TCEP.

### Synthesis of MS2042

#### General Chemistry Procedures

All reagents were purchased from Sigma-Aldrich or Fisher Scientific. Solvents used in reactions were dried before use unless otherwise noted. Analytical HPLC – Method A: data were acquired using an Agilent 6110 series system with UV detection at 254 nm. Column: Agilent Eclipse Plus 4.6 mm × 50 mm, 1.8 um C_18_ column. Solvents: A: 0.1% acetic acid in water; B: 0.1% acetic acid in methanol. Gradient: 10% to 100% B over 5.0 min, followed by 100% B for 2 min, at 1.0 mL/min. Method B: data were acquired using an Agilent Zorbax 300SC-C18 (5 μm) column with UV detection at 254 nm on an Agilent 1200 Series LC/MSD TOF machine. Solvents: A: 0.1% acetic acid in water; B: 0.1% acetic acid in methanol. Gradient: 1% B for one minute, 1 to 100% B over 3.0 min, followed by 100% B for 4 min, at 1.0 mL/min. Low resolution mass spectrometry (LRMS) data were acquired in positive ion mode on an Agilent 6110 single quadrupole mass spectrometer with electrospray ionization. Nuclear magnetic resonance (NMR) spectra were recorded on a Varian Mercury spectrometer at 400 MHz for proton (^1^H NMR) and 100 MHz for carbon (^13^C NMR). In other cases, NMR were recorded on a Bruker DRX spectrometer at 600 MHz for proton (^1^H NMR) and 150 MHz for carbon (^13^C NMR). Chemical shifts are reported in ppm (δ). Preparative HPLC was performed on an Agilent Prep 1200 series with UV detector set to 254 nm, using a Phenomenex Luna 75 mm × 30 mm, 5 um C_18_ column, with a flow rate of 30 mL/min. High resolution mass spectrometry (HRMS) data was acquired on an Agilent 1200 Series LC/MSD TOF. Medium pressure liquid chromatography (MPLC) was performed on a Combiflash Isco machine. Final compounds had >95% purity as judged by analytical HPLC unless noted.

#### Synthesis scheme of MS2042 ((*S*)-2-amino-4-((((2*R*,3*S*,4*R*,5*R*)-5-(6-amino-9*H*-purin-9-yl)-3,4-dihydroxytetrahydrofuran-2-yl)methyl)(4-fluorobenzyl)amino)butanoic acid)

6-Amino-N6,N6-bis(tert-butoxycarbonyl)-9-[5,6-dideoxy-6-(ethoxysulfonyl)-2,3-O- isopropylidene-β-D-ribo-hex-5-enofuranosyl]-9H-purine was prepared as described^22^. 6-Amino-N6,N6-bis(tert-butoxycarbonyl)-9-[5,6-dideoxy-6-(ethoxysulfonyl)-2,3-O-isopropylidene-β-D-ribo-hex-5-enofuranosyl]-9H-purine (350 mg, 0.86 mmol) and N-α-Boc-L-2,4-diaminobutyric acid tert-butyl ester hydrochloride (249 mg, 0.8 mmol) were stirred in 10 mL MeOH for 3 hours and then evaporated to dryness. The solid was dissolved in 20 mL of 1,2-dichloroethane, treated with acetic acid (60 μL) and sodium triacetoxy borohydride (212 mg, 1 mmol). This was stirred for 24 hours than diluted with saturated aqueous sodium bicarbonate. The product was extracted with dichloromethane, washed with brine, and purified on silica gel (gradient of 0 to 50% ethyl acetate in hexane). HRMS revealed the mono-alkylated product. HRMS (ESI-TOF): [M + H]^+^ Expected: 764.4194; Found: 764.4209. The resulting solid (60 mg) was dissolved in MeOH (2 mL) and treated with: 4-fluoro-benzaldehyde (12.5 μL, 0.12 mmol), acetic acid (7.2 μL, 0.12 mmol), and sodium cyanoborohydride (8 mg, 0.12 mmol). This was stirred for 24 hours and then treated with saturated aqueous sodium bicarbonate. The product was extracted with dichloromethane, and the combined organic extracts were dried to a residue. This residue was treated with acetonitrile (1.5 mL), water (0.5 mL) and trifluoroacetic acid (1.5 mL) and stirred for 2 hours. Purification by HPLC gave the product as a trifluoroacetic acid salt. Yield: 15 mg; (0.025 mmol, 3% over three steps) HRMS (ESI-TOF): [M + H]^+^ Expected: 476.2058; Found: 476.2066. ^1^H NMR (400 MHz, MeOD, Supplementary Fig. 4) δ 8.38 (s, 1H), 8.31 (s, 1H), 7.50 (m, 2H), 7.11 (m, 2H), 6.12 (s, 1H), 4.63 (d, 1H), 4.46 (m, 3H), 4.30 (d, 1H), 3.84 (s, 1H), 3.52 (m, 3H), 2.34 (s, 1H), 2.13 (s, 1H). 13C NMR (100 MHz, CDCl_3_) δ 170.76, 164.18, 162.53, 161.13, 152.41, 148.27, 142.27, 132.91, 126.29, 119.57, 115.64, 115.49, 90.80, 78.86, 73.22, 72.15, 56.82, 54.38, 52.11, 51.67, 25.04.

### Isothermal titration microcalorimetry

Experiments were performed with MicroCal iTC_200_ (GE Healthcare). Ligand SAM/SAH/MS2042 was loaded in the syringe and titrated into 60 μM of Apo ZIKV NS5-MTase in the cell. Care was taken to ensure buffer match for the ligand and ZIKV NS5-MTase to eliminate heat from buffer mismatch. The initial concentration used was 600 μM for SAM and SAH and 1 mM for MS2042. Titrations were performed at 25 °C with the standard 10 μcal/s reference power. Data were integrated, analyzed using the MicroCal iTC_200_ Analysis Origin software provided with the instrument. All three ligands displayed 1:1 binding (within model error) and sigmoidal binding isotherms, suggestive of exothermic binding. The equilibrium dissociation constant^23^ *K*_D_ for each ligand is shown in Table 1.

### Crystallization and structure determination

To obtain co-crystals of ZIKV NS5-MTase and MS2042, a mixture was prepared by adding 10 fold molar excess of the ligand to Apo ZIKV NS5-MTase. The mixture was incubated on ice for ~15 minutes and initial screens were set up with the Oryx robot at 20 °C. The best looking crystals were obtained in conditions containing PEG 4000, 2-propanol, glycerol and sodium citrate trihydrate tribasic. For data collection, crystals were cryoprotected by quick dipping in perfluoropolyether cryo oil (Hampton Research) and flash-cooled in liquid nitrogen. Diffraction data were collected at the Advanced Photon Source (beamline 23-ID-D) under cryogenic conditions at a wavelength of 1.03324 Å, and indexed with HKL2000^24^.

The ZIKV NS5-MTase_MS2042_ structure was solved by molecular replacement using the Auto-Rickshaw web server (http://webapps.embl-hamburg.de/cgi-bin/Auto-Rick/arinitAR1.cgi)^25^ and contains two molecules in the asymmetric unit. Model obtained from the Auto-Rickshaw pipeline was improved by iterative manual building and refinement with Coot^26^ and Phenix^27^ respectively. After the protein chain was built, significant difference electron density (2.5σ) was visible in the SAH/SAM binding site (Fig. 2c). The electron density was consistent with the presence of MS2042 ligand in both the chains of the asymmetric unit. This difference density was modeled as MS2042. The slightly lower occupancy of the fluorine atom and the slightly different orientation of the 4-fluorobenzyl moiety of MS2042 bound to the two chains is suggestive of conformational flexibility in binding. The structure of NS5-MTase_MS2042_ was refined to 1.55 Å (R_free_ of 19.9%; R_work_ of 17.3%). The model has excellent stereochemistry as shown by MolProbity^28^ with 99.5% of all residues in allowed regions of the Ramachandran plot and 0.5% in the disallowed regions. The residues in the disallowed regions include Asp256 from Chain A and Glu255 and Asp256 from chain B. These residues constitute a stretch of amino acids (253–258) that mediate interactions between the two molecules of the asymmetric unit and have disordered density.

### Modeling

The model of ZIKV NS5-MTase_MS2042_ bound to RNA was obtained by using the x-ray structure of full length DENV3 NS5 bound to an octameric RNA harboring a cap-0 (^N7Me^Gppp) moiety at its 5′ end (PDB ID: 5DTO)^13^. The structures were superimposed via the NS5-MTase domains using PyMol (https://www.pymol.org/). ZIKV and DENV NS5-MTase share high sequence similarity and superimpose with a r.m.s. deviation of 0.46 Å for 210 Cα atoms.

MS2042 was modeled in the human mRNA nucleoside-2′-O methyltransferase (human CMTr1, 4N49) and mRNA cap guanine-N7 methyltransferase (human RNMT, 3EPP) by first superimposing the proteins via the Rossmann fold and then optimizing the alignment so good overlap was achieved between MS2042 bound to ZIKV NS5-MTase and SAM/sinefungin in the structure of human CMTr1/RNMT (Supplementary Fig. 3).Qualiative electrostaic surface potentials and figures were prepared using PyMol﻿.

## Electronic supplementary material

Supplementary Information
