# Supplementary material for: Development of a S-adenosylmethionine analog that intrudes the RNA-cap binding site of Zika methyltransferase
Source: Sci Rep. 2017 May 9;7:1632. doi: 10.1038/s41598-017-01756-7 (PMC5431627; doi:10.1038/s41598-017-01756-7)
Supplement: Supplementary file 1 — Supplementary Information [file 41598_2017_1756_MOESM1_ESM.pdf]

Supplementary Information

Development of a S-adenosylmethionine analog that intrudes the RNA-cap binding site of Zika methyltransferase

Rinku Jain[1], Kyle V. Butler[1], Javier Coloma[1], Jian Jin[1] and Aneel K. Aggarwal[1¶]

[1]Department of Pharmacological Sciences

Icahn School of Medicine at Mount Sinai

1425 Madison Avenue

New York, New York, USA

Supplementary Table 1: **Data collection and refinement statistics**

	ZIKV NS5-MTase$_{MS2042}$
**Data collection**	
Space group	P 2(1)
Cell dimensions	
a, *b*, *c* (Å)	38.99, 111.23, 77.40
α, *β*, *γ* (°)	90.0, 93.9, 90.0
Resolution (Å)	50 - 1.55 (1.58 - 1.55) [a]
[b] R_{sym}	0.069 (0.601)
$I/\sigma(I)$	23.1 (2.4)
Completeness (%)	99.9 (97.8)
Redundancy	6.3 (3.3)
**Refinement**	
Resolution (Å)	35.7-1.55
No. reflections	95009
[c,d] R_{work} / R_{free}	0.173 / 0.199
No. atoms	
Protein	4183
MS2042 / others	68 / 42
Water	601
B factors (Å^2)	
Protein	27.4
MS2042/others	23.7 / 42.4
Water	40.9
R.m.s. deviations	
Bond lengths (Å)	0.006
Bond angles (°)	0.845

[a]Values in parentheses are for highest-resolution shell.
[b] $R_{sym} = \Sigma|I-<I>|/\Sigma I$, where I is the integrated intensity of a given reflection.
[c] $R_{cryst} = \Sigma||F_{observed}| - |F_{calculated}||/\Sigma|F_{observed}|$
[d]For R_{free} calculations, 5 % of data excluded from refinement were used

a Synthesis of MS2042

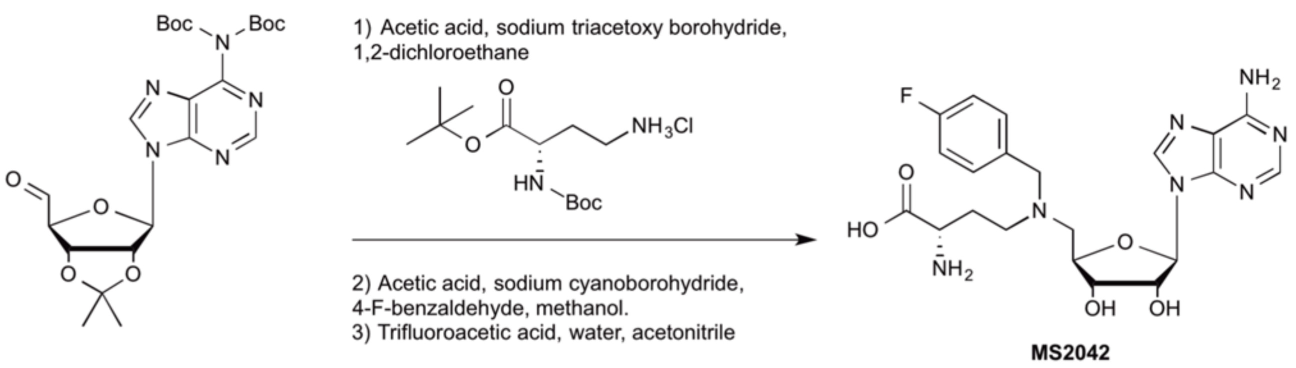

b Size exclusion profile for ZIKV NS5-MTase

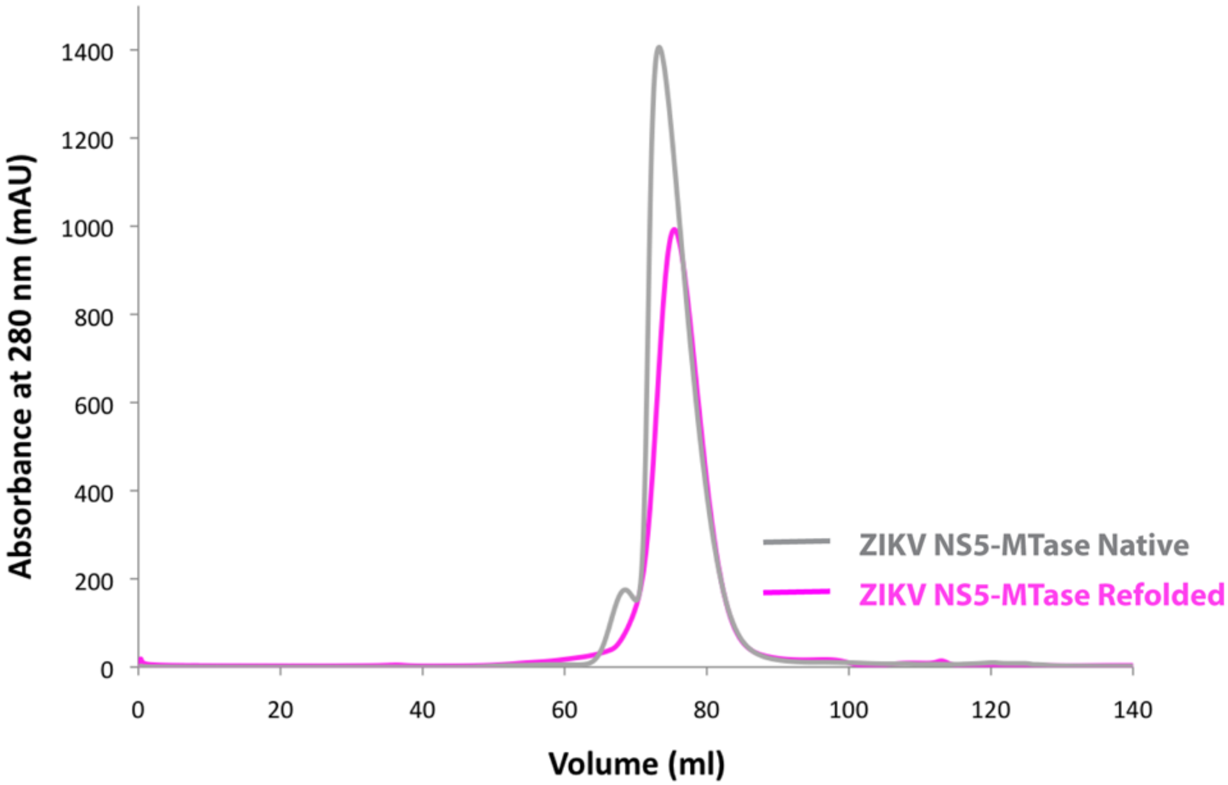

Supplementary Figure 1

(a) Synthesis scheme for MS2042. (b) Size exclusion chromatography traces for ZIKV NS5-MTase$_{SAM}$ and refolded NS5-MTase. ZIKV NS5-MTase$_{SAM}$ was denatured in 8 M urea to remove endogenous SAM/SAH, and then refolded and purified by dialysis and size exclusion chromatography.

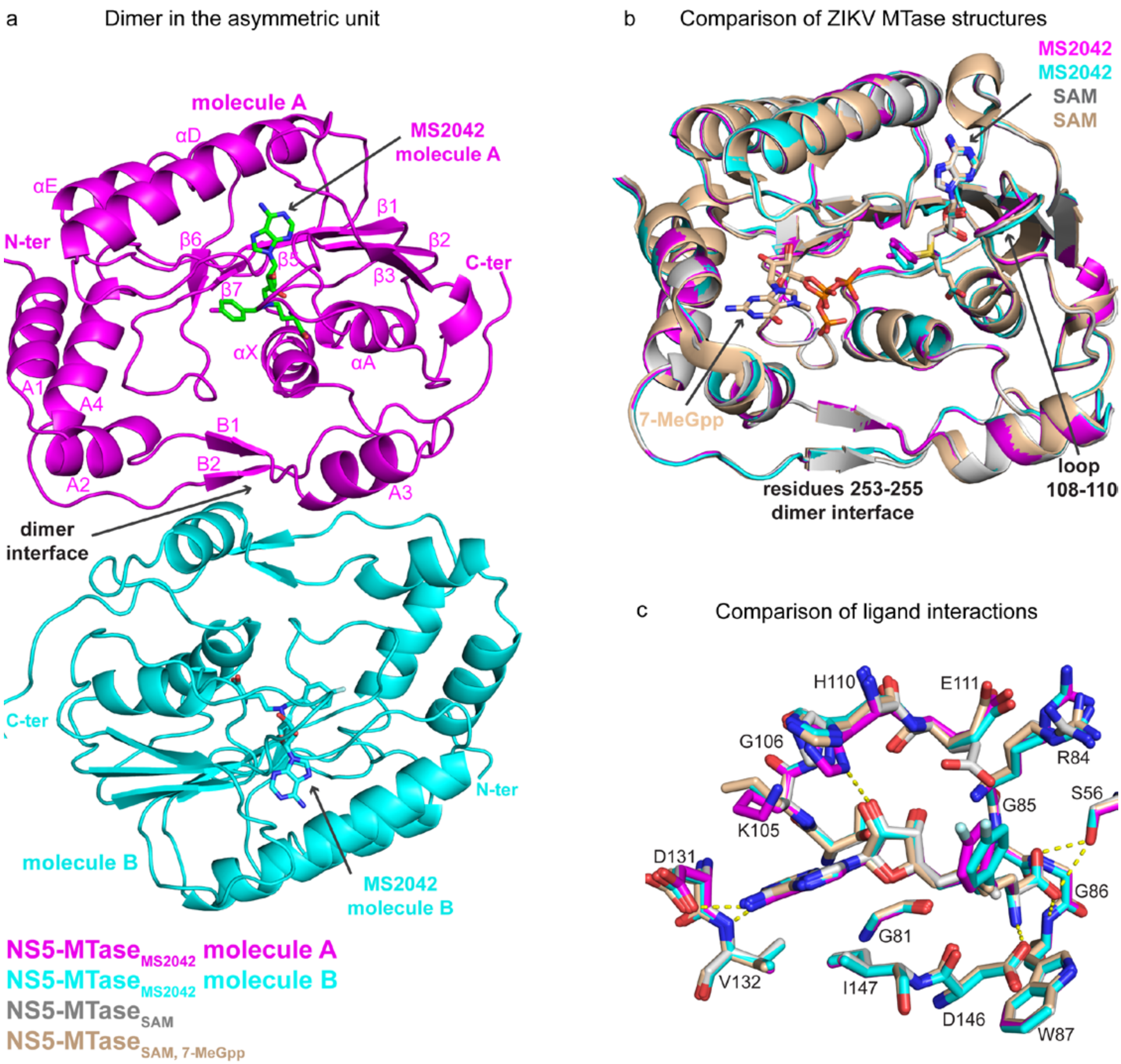

Supplementary Figure 2

Contents of the asymmetric unit and comparison of ZIKV MTase structures. (a) The asymmetric unit of ZIKV NS5-MTase$_{MS2042}$ has two protein molecules (b) The two protein chains are nearly identical to each other and to ZIKV NS5-MTase$_{SAM}$ (gray) and NS5-MTase$_{SAM,7\text{-}MeGpp}$ (tan) (c) Comparison of SAM/MS2042 interactions. Solvent mediated interactions have been omitted for clarity. Direct hydrogen bonding interactions between MS2042 and the ZIKV NS5-MTase are shown as dashed lines

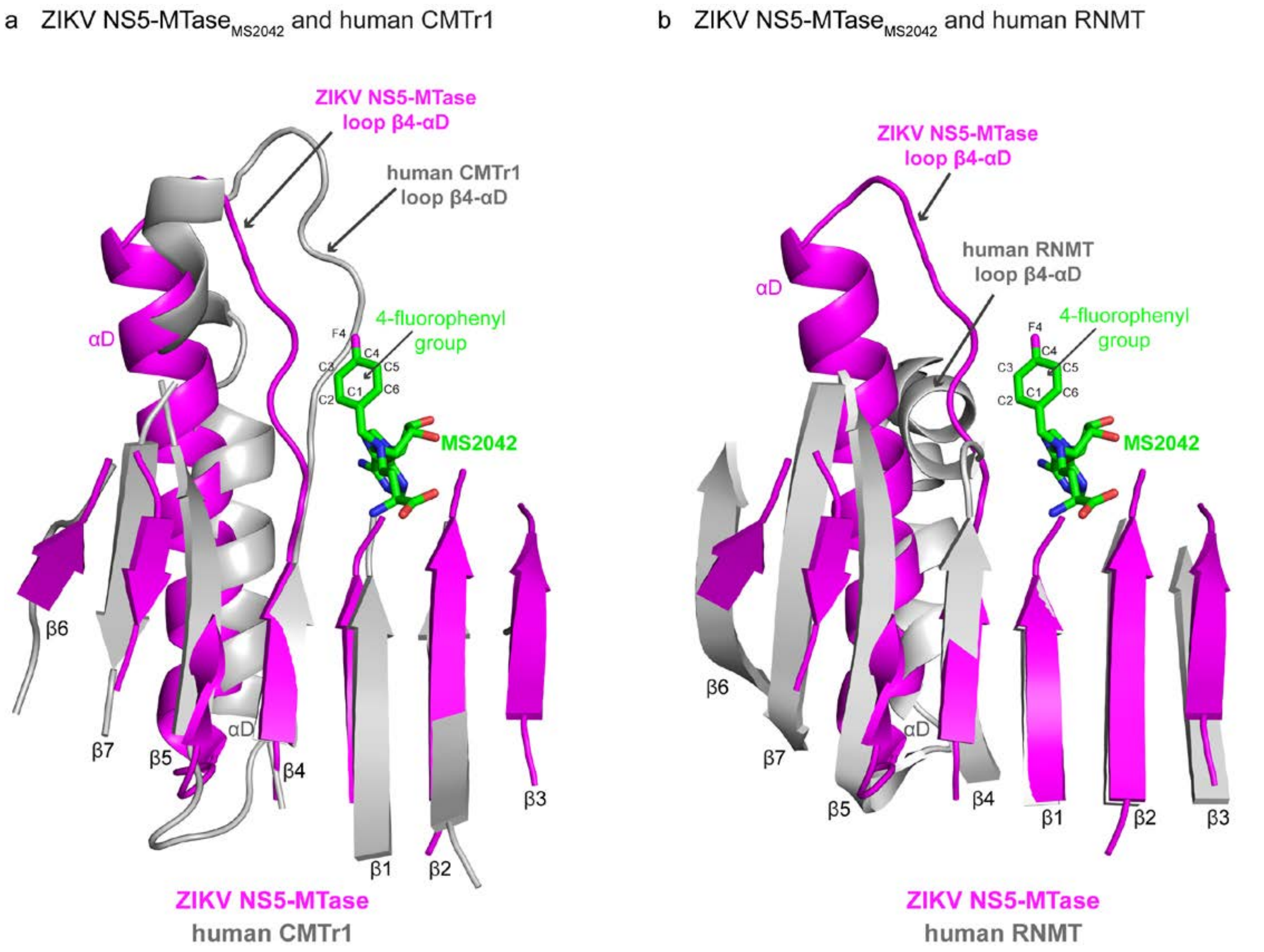

Supplementary Figure 3

Superimposition of ZIKV NS5-MTase$_{MS2042}$ and human mRNA methyltransferases.

Comparison of ZIKV NS5-MTase$_{MS2042}$ with (a) human mRNA nucleoside-2'-O methyltransferase (CMTr1, PDB id 4N49), and (b) human mRNA cap guanine-N7 methyltransferase (RNMT, PDB id 3EPP). MS2042 (green) was modeled into CMTr1 and RNMT by superimposing the seven-stranded Rossmann fold of each with that of ZIKV NS5-MTase$_{MS2042}$ such that good agreement was obtained between ZIKV NS5-MTase bound MS2042 and SAM/sinefungin in CMTr1 (4N49) and RNMT (3EPP) respectively. The β4-αD loop is labeled.

MS2042

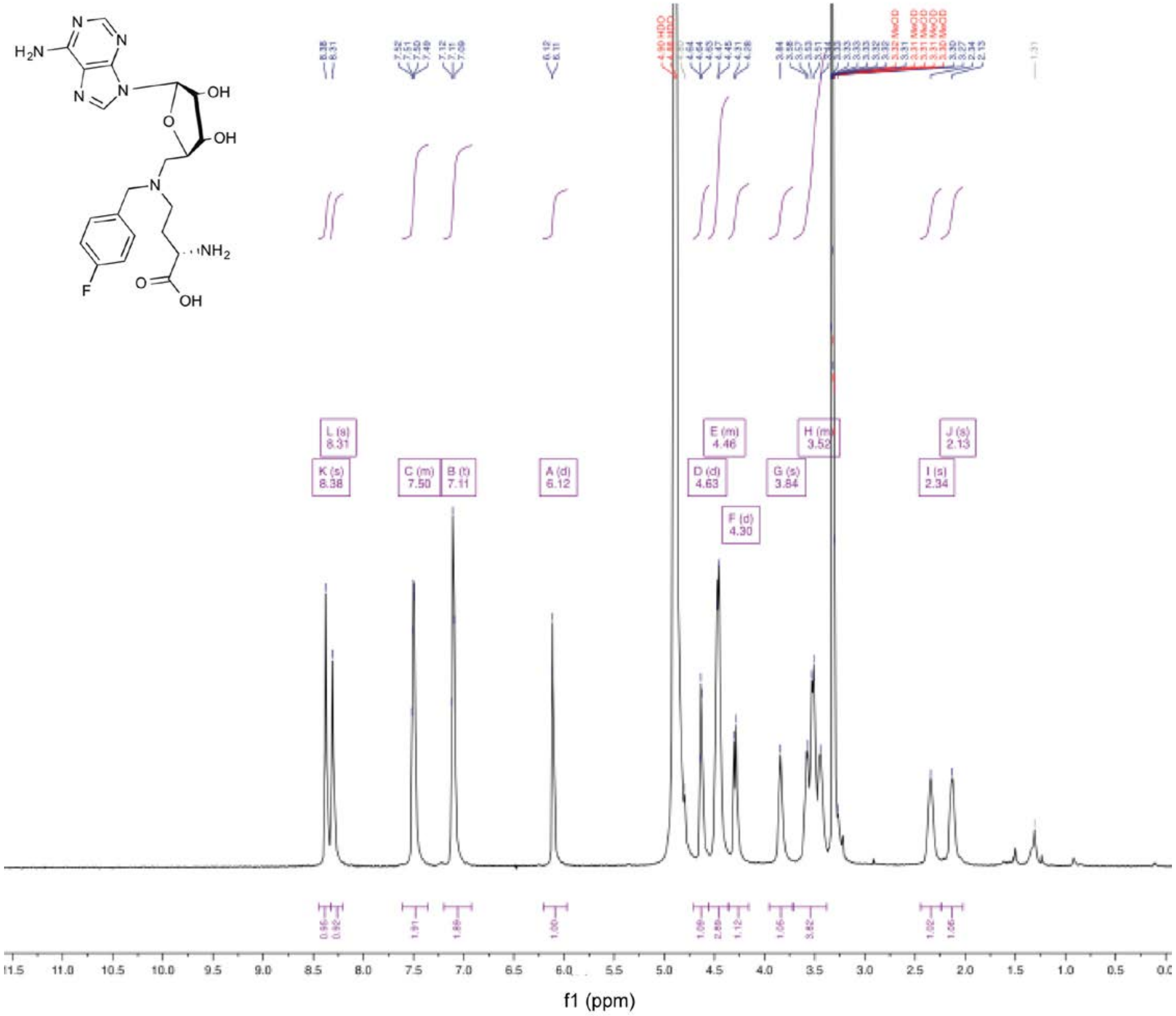

Supplementary Figure 4

^{1}H NMR spectrum of MS2042
